# The use of gamma-irradiation and ultraviolet-irradiation in the preparation of human melanoma cells for use in autologous whole-cell vaccines

**DOI:** 10.1186/1471-2407-8-360

**Published:** 2008-12-04

**Authors:** Donna H Deacon, Kevin T Hogan, Erin M Swanson, Kimberly A Chianese-Bullock, Chadrick E Denlinger, Andrea R Czarkowski, Randy S Schrecengost, James W Patterson, Mark W Teague, Craig L Slingluff

**Affiliations:** 1 Department of Surgery, University of Virginia, Charlottesville, VA, 22908, USA; 2 Human Immune Therapy Center, University of Virginia, Charlottesville, VA, 22908, USA; 3 Department of Pathology, University of Virginia, Charlottesville, VA 22908, USA

## Abstract

**Background:**

Human cancer vaccines incorporating autologous tumor cells carry a risk of implantation and subsequent metastasis of viable tumor cells into the patient who is being treated. Despite the fact that the melanoma cell preparations used in a recent vaccine trial (Mel37) were gamma-irradiated (200 Gy), approximately 25% of the preparations failed quality control release criteria which required that the irradiated cells incorporate ^3^H-thymidine at no more than 5% the level seen in the non-irradiated cells. We have, therefore, investigated ultraviolet (UV)-irradiation as a possible adjunct to, or replacement for gamma-irradiation.

**Methods:**

Melanoma cells were gamma- and/or UV-irradiated. ^3^H-thymidine uptake was used to assess proliferation of the treated and untreated cells. Caspase-3 activity and DNA fragmentation were measured as indicators of apoptosis. Immunohistochemistry and Western blot analysis was used to assess antigen expression.

**Results:**

UV-irradiation, either alone or in combination with gamma-irradiation, proved to be extremely effective in controlling the proliferation of melanoma cells. In contrast to gamma-irradiation, UV-irradiation was also capable of inducing significant levels of apoptosis. UV-irradiation, but not gamma-irradiation, was associated with the loss of tyrosinase expression. Neither form of radiation affected the expression of gp100, MART-1/MelanA, or S100.

**Conclusion:**

These results indicate that UV-irradiation may increase the safety of autologous melanoma vaccines, although it may do so at the expense of altering the antigenic profile of the irradiated tumor cells.

## Background

Cellular immune responses to autologous tumor cells have been documented in cancer patients including those with melanoma. Antigens recognized by tumor-specific T cells have been categorized as cancer-testis antigens, differentiation proteins, mutated gene products, widely expressed proteins, and viral proteins [1-3]. Vaccines incorporating synthetic forms of these antigens may be immunogenic, but the ensuing immune response can only be effective if the tumor in the vaccine recipient expresses one or more of the antigens present in the vaccine. This can be problematic because cancer-testis antigens are expressed only in a subset of tumors [4,5]. and differentiation antigens are often down-regulated in metastases [6-11]. Thus, peptide, protein, or DNA-based vaccines currently being tested can potentially stimulate immune responses for which there is no target in a particular patient. Further, such vaccines designed for use in a general population do not contain unique antigens arising from mutated gene products as these antigens would only be useful in the patient whose tumor expresses them [12-14]. Targeting unique antigens might prove advantageous, however, as many of the altered proteins may play a role in the malignant phenotype of the cell [13,14].

An ideal synthetic vaccine would contain each of the antigens expressed by the tumor cells of an individual patient, however, with the limitations of current antigen identification technology this is not yet feasible. Until antigen identification technology can be performed rapidly on a customized basis for each patient, approaches to vaccination with unique tumor antigens or otherwise undiscovered antigens will require incorporation of the autologous tumor tissue in the vaccines. Such approaches include vaccination with autologous tumor cells [15,16], RNA derived from autologous tumor cells [17,18], or heat shock proteins derived from autologous tumor cells [19,20]. Autologous tumor cells may be administered as viable cells alone [15,16], as viable cells with dendritic cells (DC) [21], or as cell lysates added to DC [22,23].

A concern for patient safety with autologous tumor cell vaccines is that viable autologous tumor cells could proliferate and metastasize in the host. To prevent this from occurring after vaccination, a standard approach used in human clinical trials has been to pre-treat the tumor cells with 25 to 200 Gy of gamma irradiation [24-29]. We have enrolled patients in one such melanoma vaccine trial using autologous tumor cells (Mel37). To provide optimal patient safety within this trial, the tumor cells were gamma-irradiated (200 Gy) prior to vaccination. As part of the quality assurance release criteria, a ^3^H-thymidine uptake assay was then performed to ensure that the irradiated tumor incorporated ^3^H-thymidine at no more than 5% of the level found in the non-irradiated tumor.

Our experience with the Mel37 trial has been useful in establishing additional guidelines and procedures to help ensure the safety of autologous tumor cell vaccines. In particular, we demonstrate the resistance of some patients' metastatic tumor cells to 200 Gy gamma-irradiation as demonstrated by the ability to incorporate ^3^H-thymidine despite being given that dose of radiation. We therefore investigated ultraviolet (UV) radiation for its ability to block ^3^H-thymidine uptake and to induce apoptosis of tumor cells. The results from this study demonstrate that the combination of gamma-irradiation and UV-irradiation was found to give the best control of tumor cell proliferation in vitro.

## Methods

### Tumor tissue collection and human subjects approval

All research involving human subjects and human tissues was approved by the University of Virginia Institutional Review Board (IRB# 8577) in accordance with an assurance filed with and approved by the Department of Health and Human Services (BB-IND# 8932). Tumor specimens were obtained through the Tissue Procurement Facility of the University of Virginia.

### Tumor cell preparation

Tumor specimens collected sterilely from the operating room were cut into 2–8 mm thick slices and immersed in Hanks' balanced salt solution (HBSS, Life Technologies, Grand Island, NY). The tumor specimen was mechanically dissociated with a sterile scalpel. Remaining tumor fragments were transferred to a 50 ml conical tube with RPMI-1640 (Life Technologies) containing 1 mg/ml collagenase (Worthington Biochemical Corp., Lakewood, NJ), 10 μg/ml DNAase (Worthington Biochemical Corp.), 2.5 U/ml hyaluronidase (Worthington Biochemical Corp.), 100 μg/ml penicillin G (sodium salt), 100 μg/ml streptomycin sulfate, 0.25 μg/ml amphotericin B, and 5% autologous serum or human AB serum (Sigma Chemical Co., St. Louis, MO) and incubated at room temperature. Dissociated tumor cells were washed by centrifugation with HBSS. Cell counts with trypan blue were performed to determine tumor cell viability and yield. Tumor cells were cryopreserved in 10% DMSO, 90% autologous serum or human AB serum using a controlled rate freezer.

### Assay medium

RPMI-1640 was supplemented with 10% fetal bovine serum, 2 mM L-glutamine, 100 U/ml penicillin, and 100 μg/ml streptomycin (complete RPMI).

### Gamma irradiation of tumor cell suspensions

A Gammacell 3000 Elan (MDS Nordion, Ottawa, ON, Canada) with a Cesium-137 source used for gamma irradiation. Tumor cell suspensions at 1–5 × 10^6 ^cells/ml in complete RPMI were irradiated at 555.5 Gy/min.

### Ultraviolet irradiation of tumor cell suspensions

Cell suspensions were plated into 6-well plates at 2–4 × 10^6 ^cell/ml in a total volume of 1.5 ml of complete RPMI. The plate was placed on a UV transilluminator box (UVP, Inc., Upland, CA) and exposed to a combination of UVA (84 mJ/cm^2^/min) and UVB (26 mJ/cm^2^/min) for 15 sec to 10 min. A UVA monitor (UVA-400C, National Biological Corp., Twinsburg, OH) and UVB monitor (UVB-500C, National Biological Corp.) were used to measure the UV dose rate.

### Combination ultraviolet and gamma irradiation of tumor cells

Cells were first exposed to UV irradiation and then exposed to gamma irradiation as described above.

### Assay of ^3^H-thymidine incorporation after irradiation

Tumor cell suspensions in complete RPMI were plated in 5–6 replicates at 20,000–50,000 cells/200 μl in flat-bottom 96-well plates (Costar, Lowell, MA) and incubated at 37°C in a humidified, 5% CO_2 _incubator for 5 days. One μCi of ^3^H-thymidine in 25 μl of complete RPMI was then added to each well and the incubation continued for an additional 18–24 h. Cells were then harvested using a MACH IIIM Harvester 96 (Tomtec, Hamden, CT) and the amount of incorporated ^3^H-thymidine in counts per minute (CPM) was determined using a 1450 MicroBeta Trilux Liquid Scintillation and Luminescence Detector (PerkinElmer, Boston, MA). Background ^3^H-thymidine incorporation was initially determined with wells containing only complete medium while later experiments used wells containing a comparable number of cells that had been subjected to three freeze-thaw cycles (freezing in liquid nitrogen followed by thawing in a 37°C water bath). As a control, the lymphoblastoid cell line K562 was treated in a similar fashion. Incorporated ^3^H-thymidine was determined as: CPM_cells _- CPM_background_. Percent maximal ^3^H-thymidine incorporation was determined as: [(CPM_irradiated _- CPM_background_)/(CPM_non-irrdiated _- CPM_background_)] × 100.

### Apoptosis assays

Caspase-3 activity was measured as previously described using DEVD-AFC (Calbiochem, San Diego, CA) as the substrate [30]. DNA fragmentation was measured using a Cell Death Detection ELISA^PLUS ^kit (Roche, Indianapolis, IN) according to the manufacturer's instructions.

### Immunohistochemistry

To evaluate antigen expression at various time points after irradiation, tumor cell suspensions were pelleted by centrifugation. Cell blocks were made by pelleting the cells, fixing them with 10% formalin, and then embedding them in paraffin. Immunohistochemistry stains were performed by the Pathology Department using clinical-grade antibodies to gp100 (HMB45, Dako, Carpinteria, CA), tyrosinase (T311, Vector Laboratories, Burlingame, CA), MART-1/MelanA (A103, Dako), and S100 (Dako) and the slides were read by a pathologist (MWT).

### Western blot analysis

Cells were harvested and lysates prepared using NP-40 lysis buffer [31]. Protein concentration was determined using a BCA Protein Assay Kit (Pierce, Rockford, IL). Proteins (40 μg/lane) were resolved on 4–12% Bis-Tris polyacrylamide gels and then transferred to PVDF membranes (Pierce, Rockford, IL). After blocking with 1% bovine serum albumin, the membranes were probed with a rabbit polyclonal antisera to tyrosinase (clone H-109, Santa Cruz Biotechnology, Santa Cruz, CA) and a mouse monoclonal antibody to GAPDH (clone 6C5, Millipore, Billerica, MA). Peroxidase-linked sheep anti-mouse and donkey-anti-rabbit antisera (Amersham Biosciences, Piscataway, NJ) in conjunction with the SuperSignal West Pico Chemiluminescent substrate (Pierce) were used to detect binding of the primary antibodies.

## Results

### Proliferation of gamma-irradiated melanoma cells prepared for autologous vaccination

Mel37 was an autologous melanoma vaccine trial conducted at the University of Virginia (UVA). To minimize the possibility of outgrowth of the tumor cells in the vaccine recipient, the tumor cells were gamma irradiated (200 Gy) prior to administration. The irradiated tumor cells were then tested for their ability to incorporate ^3^H-thymidine in comparison to the non-irradiated control sample. As part of the lot vaccine release criteria, the ^3^H-thymidine incorporation of the irradiated cells had to be less than 5% of the ^3^H-thymidine incorporation of the corresponding non-irradiated cells. Vaccine preparations that failed to meet those criteria were not used.

**Figure 1:**
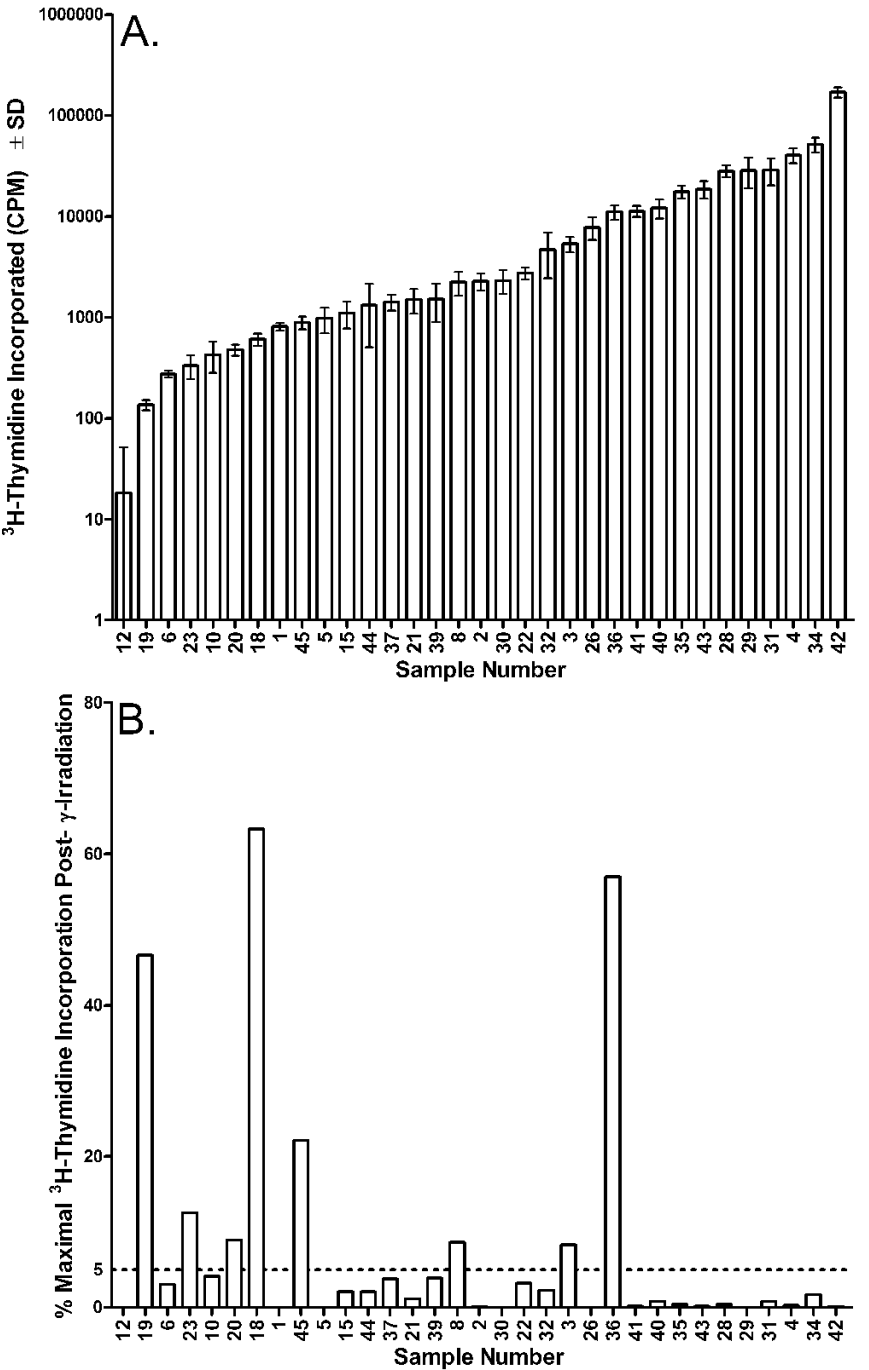
**^3^H-thymidine incorporation of melanoma cells prior to and following treatment with 200 Gy gamma-irradiation**. Human melanoma deposits were resected surgically and the tumors rendered into single cell suspensions by mechanical and enzymatic dissociation. The cell suspensions were then prepared into a tumor cell vaccine and split into samples receiving or not receiving 200 Gy gamma-irradiation. The non-irradiated and irradiated cells were then cultured *in vitro *for five days prior to the addition of ^3^H-thymidine for 18 to 24 hr. (A) Proliferation in the absence of gamma-irradiation. Samples ordered from lowest to highest proliferation. (B) Proliferation following treatment with 200 Gy gamma-irradiation. The data are presented as percent maximal ^3^H-thymidine incorporation in comparison to the non-irradiated control. Samples are ordered as in (A). The dashed horizontal bar indicates 5% maximal ^3^H-thymidine incorporation.

Tumors from thirty-three melanoma patients were included in the study and demonstrated a 9,300-fold range of ^3^H-thymidine incorporation, varying from 18 to 170,014 CPM (Figure 1A). Gamma-irradiation (200 Gy) reduced the ^3^H-thymidine incorporation of twenty-five (76%) of these samples below 5% of the non-irradiated control samples (Figure 1B). The remaining eight irradiated vaccines (24%) had a mean ^3^H-thymidine incorporation of 28% (median 17%, range, 8%–63%) in comparison to the non-irradiated control samples. Notably, the ^3^H-thymidine incorporation of irradiated tumor cells from three patients exceeded 40% of the corresponding non-irradiated samples. Among the eight specimens failing the lot release criteria, five were retested from one to three times with the same results (data not shown).

The fact that 200 Gy of gamma-irradiation failed to reduce the ^3^H-thymidine incorporation of eight of thirty-three samples below 5% of the non-irradiated controls prompted us to determine if a more effective means could be found to reduce the ^3^H-thymidine incorporation of the treated samples. These studies, which follow, were conducted with melanoma cell lines rather than fresh patient samples, as cells from the lines are not limiting in numbers.

### Dose response of gamma-irradiation on the proliferative capacity of melanoma cell lines

**Figure 2:**
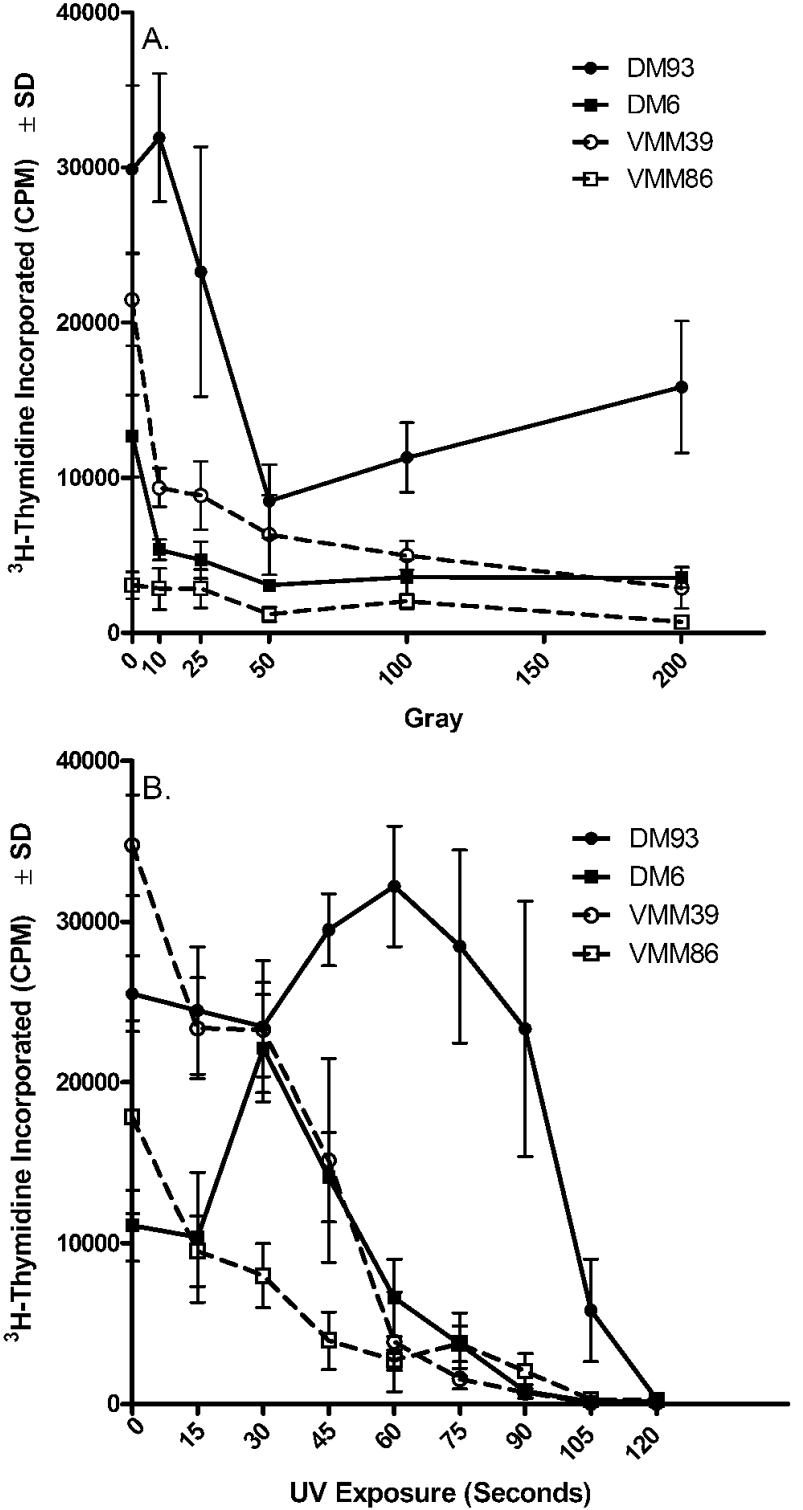
**Effect of gamma-irradiation and UV-irradiation dose titration on the ^3^H-thymidine incorporation of melanoma cell lines**. Melanoma cell lines, either untreated or treated with the indicated dose of (A) gamma-irradiation or (B) UV-irradiation, were cultured *in vitro *for five days prior to the addition of ^3^H-thymidine for 18 to 24 hr.

The inability of 200 Gy of gamma-irradiation to decrease ^3^H-thymidine incorporation of melanoma cells to less than 5% of that seen in the corresponding non-irradiated controls could be the result of using too low a dose of irradiation, although the dose chosen is at the upper limit of what has been used in other studies [24-29]. A gamma-irradiation dose response experiment with four different melanoma cell lines was therefore conducted to determine if 200 Gy is a reasonable dose. The results indicate that ^3^H-thymidine rapidly decreased through about 50 Gy and then remained at a low, but measurable level through 200 Gy (Figure 2A). In no case, however, did the ^3^H-thymidine of the irradiated samples relative to the non-irradiated samples decrease below 5% (DM6 = 28%, DM93 = 53%, VMM39 = 14%, VMM86 = 23%).

### Dose response of UV-irradiation on the ^3^H-thymidine uptake of melanoma cell lines

The effect of UV-irradiation on the ^3^H-thymidine uptake of melanoma cells was next tested. Adherent monolayers of cells in 6-well plates were exposed to a UV light source for varying amounts of time and at a delivery rate of 84 mJ/cm^2^/min UVA and 26 mJ/cm^2^/min UVB. Although melanoma cell line DM93 was less sensitive to lower doses of UV-irradiation than were the other three melanoma cell lines, two minutes of UV-irradiation was sufficient to reduce the ^3^H-thymidine uptake of the irradiated cells to 1.5% or less of the corresponding non-irradiated cell line (Figure 2B).

### Combined effect of gamma-irradiation and UV-irradiation on the ^3^H-thymidine uptake capacity of melanoma cell lines and fresh melanoma cells

**Figure 3:**
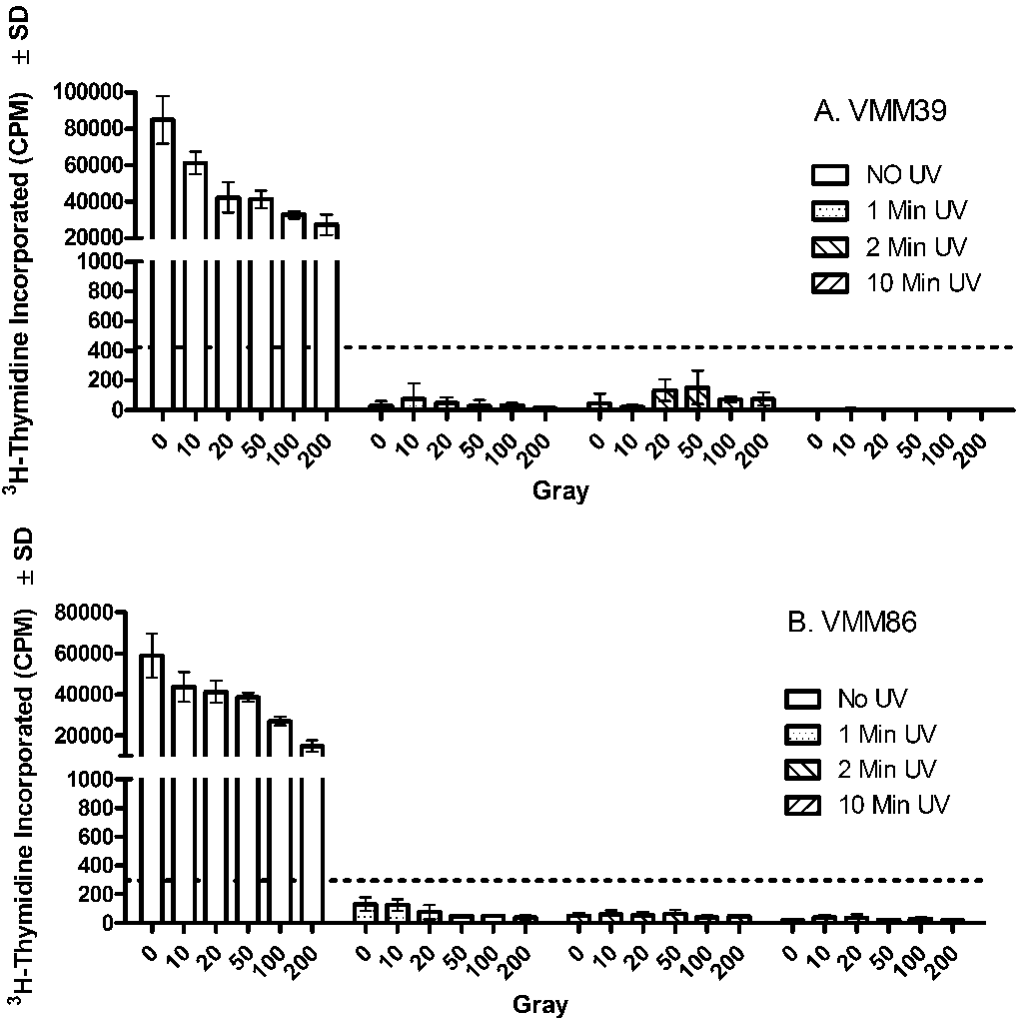
**Control of melanoma cell line ^3^H-thymidine incorporation after combination gamma-irradiation andUV-irradiation**. Melanoma cell lines (A) VMM39 and (B) VMM86 were non-irradiated or treated with varying combinations of gamma-irradiation or UV-irradiation. The cell lines were then cultured *in vitro *for five days prior to the addition of ^3^H-thymidine for 18 to 24 hr. The dashed horizontal bar indicates 5% maximal ^3^H-thymidine incorporation.

Melanoma cell lines were next subjected to 0 – 10 min of UV-irradiation followed by 0 – 200 Gy gamma-irradiation (Figure 3). As in previous experiments, gamma-irradiation alone did not reduce ^3^H-thymidine uptake below 5% of that of the non-irradiated controls. In contrast, as little as one minute of UV-irradiation was sufficient to drop the ^3^H-thymidine uptake of each of the cell lines to less than 0.5% of the non-irradiated control. Because the reduction in ^3^H-thymidine uptake with UV-irradiation was nearly complete, the combination of UV-irradiation and gamma-irradiation did not show any additive or synergistic effects within the dose ranges chosen.

**Figure 4:**
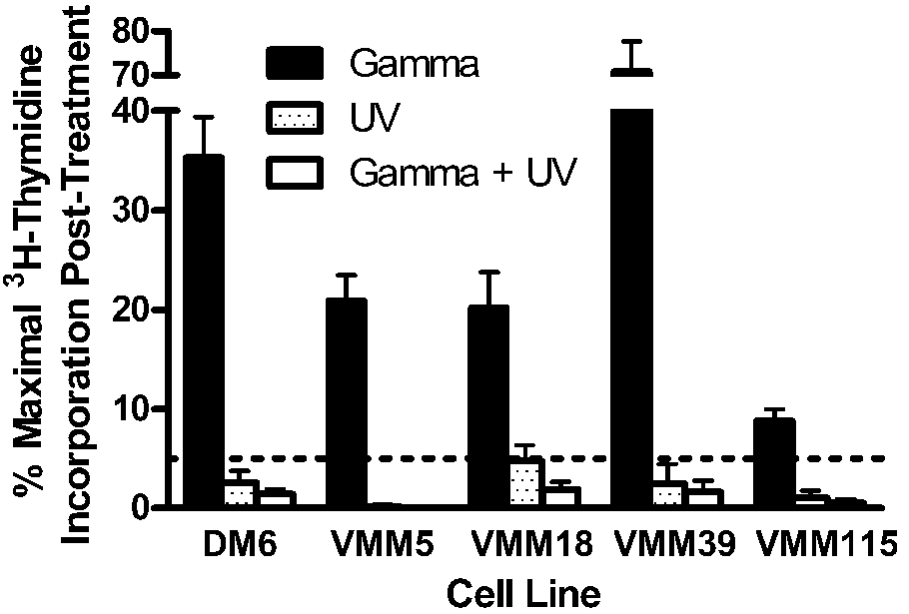
**Control of melanoma cell line ^3^H-thymidine incorporation after combination gamma-irradiation andUV-irradiation**. Melanoma cell lines were non-irradiated, gamma-irradiated (200 Gy), UV-irradiated (5 min), or both gamma-irradiated (200 Gy) and UV-irradiated (5 min). The cell lines were then cultured *in vitro *for five days prior to the addition of ^3^H-thymidine for 18 to 24 hr. The data are presented as percent maximal ^3^H-thymidine incorporation in comparison to the non-irradiated control. The dashed horizontal bar indicates 5% maximal ^3^H-thymidine incorporation.

Five melanoma cell lines were further treated with gamma-irradiation (200 Gy) alone, UV-irradiation alone (2 min), or a combination of the two (Figure 4). Relative to the non-irradiated controls, gamma-irradiation reduced the ^3^H-thymidine uptake to 31% (range = 9 – 71%), UV-irradiation reduced the ^3^H-thymidine uptake to 2.2% (range = 0.2–4.8%), and the combination of UV and gamma-irradiation reduced the ^3^H-thymidine uptake to 1.1% (range = 0.0–1.9%). Although in each case, UV-irradiation reduced the ^3^H-thymidine uptake to less than 5%, the combination of UV- and gamma-irradiation reduced ^3^H-thymidine uptake further.

**Figure 5:**
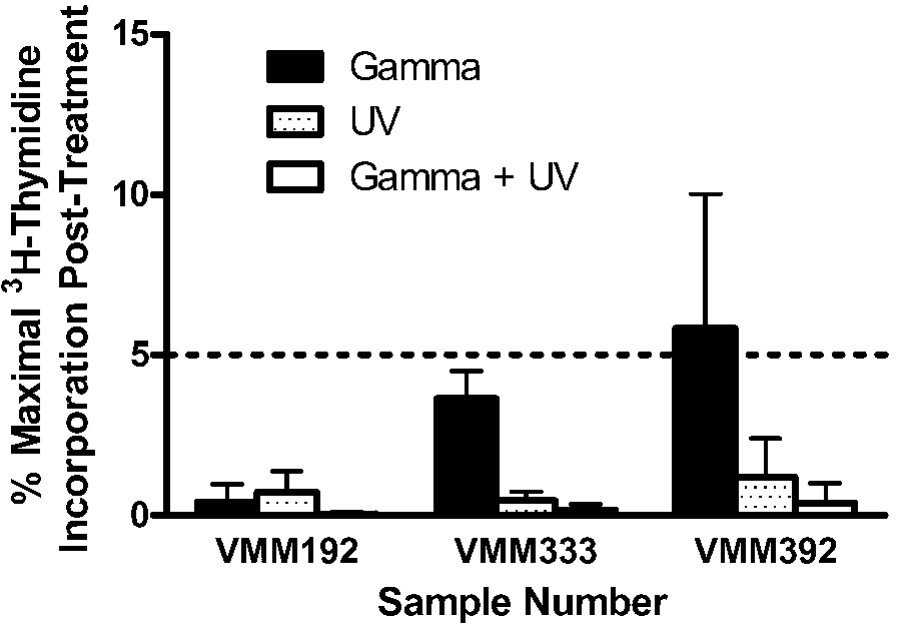
**Control of melanoma vaccine preparation ^3^H-thymidine incorporation after combination gamma-irradiation andUV-irradiation**. Melanoma cells prepared for vaccination were non-irradiated, gamma-irradiated (200 Gy), UV-irradiated (5 min), or both gamma-irradiated (200 Gy) and UV-irradiated (5 min). The cell lines were then cultured *in vitro *for five days prior to the addition of ^3^H-thymidine for 18 to 24 hr. The data are presented as percent maximal ^3^H-thymidine incorporation in comparison to the non-irradiated control. The dashed horizontal bar indicates 5% maximal ^3^H-thymidine incorporation.

Three single cell suspensions of fresh melanoma cells were also treated individually or in combination with gamma-irradiation (200 Gy) and UV-irradiation (2 min) (Figure 5). Gamma-irradiation of sample VMM392 did not reduce its ^3^H-thymidine uptake below 5% of that of the non-irradiated control, while UV-irradiation either alone or in combination with gamma-irradiation reduced its ^3^H-thymidine uptake below 5%. For each of the three samples tested, the combination of gamma-irradiation and UV-irradiation decreased ^3^H-thymidine uptake to a greater extent than did use of either form of irradiation alone.

### Effect of gamma-irradiation and UV-irradiation on the induction of apoptosis

**Figure 6:**
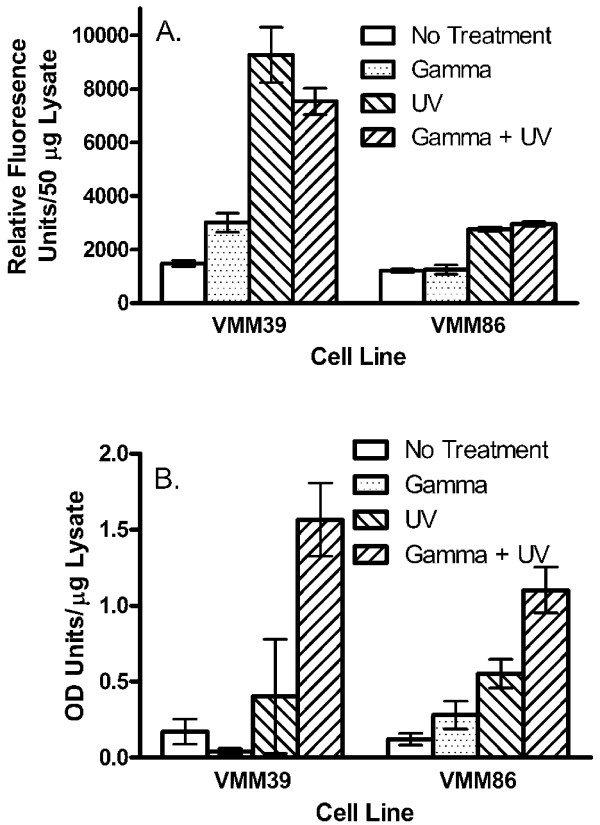
**Induction of apoptosis in melanoma cell lines following gamma-irradiation and/or UV-irradiation**. Melanoma cells prepared for vaccination were non-irradiated, gamma-irradiated (200 Gy), UV-irradiated (5 min), or both gamma-irradiated (200 Gy) and UV-irradiated (5 min). The cell lines were then cultured *in vitro *for eight hours prior to assaying (A) caspase activity or (B) DNA fragmentation.

Eight hours following treatment with gamma-irradiation, UV-irradiation or a combination of both forms of radiation, the melanoma cell lines VMM39 and VMM86 were evaluated for apoptosis by caspase and DNA fragmentation assays (Figure 6A, 6B). Gamma-irradiation by itself led to a small increase in caspase detection with melanoma line VMM39, while UV-irradiation, either alone or in combination with gamma-irradiation led to higher levels of detectable caspase with VMM39 (Figure 6A). Similar results were obtained with melanoma line VMM86 except that gamma-irradiation alone did not result in an increase in the detectable caspase. Gamma-irradiation by itself did not increase (VMM39) or only slightly increased (VMM86) DNA fragmentation over that seen in untreated controls (Figure 6B). UV-irradiation induced DNA fragmentation in both cell lines, and the combination of both types of radiation led to the largest increases in DNA fragmentation.

### Tumor viability following gamma-irradiation and UV-irradiation

**Figure 7:**
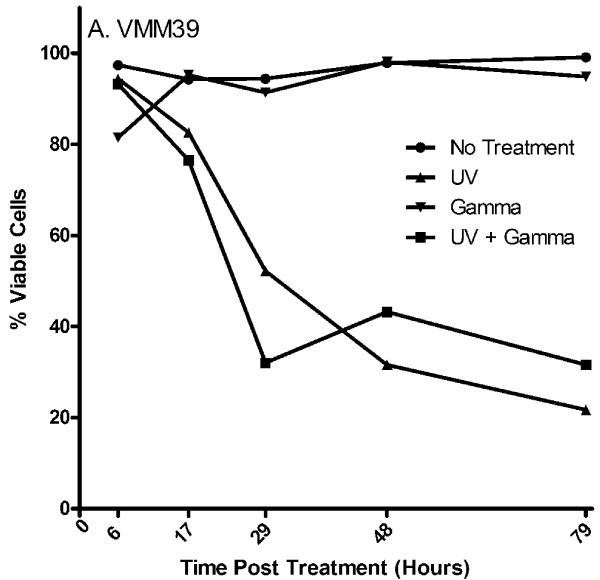
**Melanoma cell viability after irradiation**. The melanoma cell line VMM39 was evaluated by trypan blue exclusion for the persistence of viable cells at multiple time points after UV-irradiation alone (5 min), gamma-irradiation alone (200 Gy), combined UV-irradiation (5 min) and gamma-irradiation (200 Gy), or no treatment.

**Figure 8:**
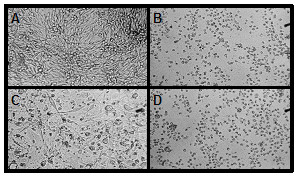
**Adherence and morphology of melanoma cell line VMM39 following gamma-irradiation and/or UV-irradiation**. VMM39 was (A) non-irradiated, (B) gamma-irradiated (200 Gy), (C) UV-irradiated (5 min), or (D) both gamma-irradiated (200 Gy) and UV-irradiated (5 min). The cell lines were then cultured *in vitro *for five days prior to microscopic evaluation. Representative areas of the cell culture surface are shown.

To determine the approximate time after radiation that human tumor cells remain viable, the melanoma cell line VMM39 was irradiated, cultured *in vitro *for up to seventy-nine hours, and evaluated for viability by trypan blue exclusion (Figure 7). Cells treated with gamma-irradiation alone remained greater than 80% viable seventy-nine hours post treatment and had comparable viability to that of the untreated cells. Conversely, cells treated with UV-irradiation or a combination of UV- and gamma-irradiation demonstrated a marked decrease in viability within 24 hours of treatment, and were less than 35% viable within seventy-nine hours following treatment. The morphology of the treated and untreated cells five days post-treatment is consistent with the observed viability (Figure 8). The VMM39 melanoma cells remained adherent five days after gamma-irradiation, but all cells had lost adherence by five days after UV-irradiation, consistent with induction of apoptosis in the vast majority of tumor cells. Treatment of the cells with both gamma-irradiation and UV-irradiation did not produce changes in morphology or adherence that were distinguishable from UV-irradiation treatment alone.

### Effect of gamma-irradiation and UV-irradiation on the stability of antigenic proteins

To evaluate if the treatment of melanoma cell lines DM6 and DM93 with gamma-irradiation and/or UV-irradiation impacts the expression of three common shared melanoma antigens (gp100, tyrosinase, MART-1) and the melanoma marker S100, the cell lines were irradiated and then cultured an additional twenty-nine hours. Cell blocks were then prepared and antigen expression determined by immunohistochemistry (Table 1). In comparison to untreated controls, the treatments, either alone or in combination, did not affect the expression of gp100, MART-1, and S100. In contrast, UV-irradiation, either alone or in combination with gamma-irradiation, rendered tyrosinase undetectable using the tyrosinase-specific antibody T311.

**Table 1:** Antigen expression in melanoma cell lines twenty nine hours following UV and/or gamma-irradiation*

	% Antigen Expression^†^							
	
	gp100		Tyrosinase		MART-1/MelanA		S100	
	
Treatment	DM6	DM93	DM6	DM93	DM6	DM93	DM6	DM93
Non-irradiated	98%	100%	25%	98%	90%	90%	100%	100%
UV	98%	100%	< 1%	5%	70%	85%	100%	100%
Gamma	100%	100%	30%	98%	80%	85%	100%	100%
UV + Gamma	98%	100%	< 1%	1%	75%	85%	100%	100%

* Melanoma cells were non-irradiated, gamma-irradiated (200 Gy), UV-irradiated (5 min), or both gamma-irradiated (200 Gy) and UV-irradiated (5 min).

^†^The percent of cells staining for gp100 expression, tyrosinase, MART-1/MelanA, and S100 expression was assessed by immunohistochemistry.

**Figure 9:**
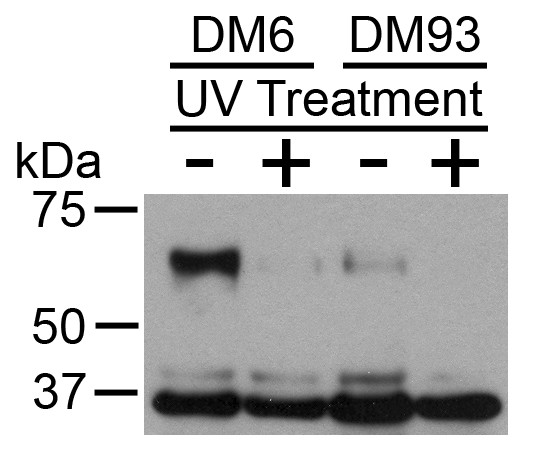
**Tyrosinase protein expression followingUV-irradiation**. Melanoma cell lines DM6 and DM93 were either non-irradiated or UV-irradiated (5 min), and then cultured an additional 29 h. Detergent lysates were then prepared and tyrosinase expression (~70 kDa) and GAPDH expression (~37 kDa) determined by Western blot analysis.

The effect of UV-irradiation on tyrosinase expression was further investigated by performing Western blot analysis (Figure 9). Cell lysates were prepared from cells treated twenty-nine hours earlier with UV-irradiation or from untreated control cells. Tyrosinase protein expression was detected in untreated DM6 and DM93 melanoma lines, with DM6 expressing higher amounts of tyrosinase than DM93. Tyrosinase protein expression was detected at significantly lower levels in the corresponding UV-irradiated cells. GAPDH expression is roughly equivalent in the different samples indicating that a similar amount of protein is present in each lane.

## Discussion

The first requirement of a new therapy is that it be safe. Although cancer vaccines are generally considered safe, vaccines incorporating autologous tumor carry a risk of implanting tumor cells that are capable of growth and dissemination in the patient. Such a risk has been demonstrated by a vast experience with transfer of tumors between syngeneic mice, but for obvious reasons, such studies have not been done in humans. There are, however, anecdotal reports that highlight the risk. These anecdotes come from older literature and involve allogeneic tumor cell transfer into patients with decreased immune competence. In one case an elderly woman was injected with her daughter's melanoma cells, which subsequently metastasized and caused her death [32]. In another case, a volunteer with impaired health developed metastases from transferred allogeneic tumor cells [33,34]. There are also documented cases of transfer of malignancy into an organ transplant recipient when the transplanted organ contained occult malignant cells [35]. These cases indicate that there is not only a theoretical risk, but also a finite risk, of tumor outgrowth in an individual receiving either autologous or allogeneic tumor cells. To guard against this risk, pretreatment of tumor cells by gamma-irradiation is a standard part of the preparation of autologous and allogeneic tumor vaccines.

There is no standard dose of gamma-irradiation with which to pre-treat tumor cells *in vitro *prior to their use in a vaccine, as previous studies have used gamma radiation ranging from 25 to 200 Gy [24-29]. *In situ*, gamma radiation is used clinically to control solid tumors, and is rarely able to destroy all tumor cells when delivered in multiple fractions to a total 50–60 Gy dose that is tolerated by surrounding tissues. With stereotactic radiation (gamma knife), high single doses in the range of 20 Gy are delivered to tumor cell deposits. This is much more effective at tumor destruction, but 20–25% of patients have tumors that fail to be controlled even after that large a dose of radiation [36,37]. Thus, there is ample evidence from clinical experience that high doses of gamma radiation may be inadequate to prevent human tumor cells from proliferating. To error on the side of caution, we chose 200 Gy of gamma-irradiation as the standard dose at which to pre-treat melanoma cells prior to use in an autologous vaccine preparation.

Following gamma-irradiation, the cells were tested for their ability to proliferate as measured by ^3^H-thymidine incorporation. The lot release criteria specified in the IND under which the Mel37 trial was conducted required that when measured between five and six days following gamma-irradiation, the ^3^H-thymidine incorporation of the irradiated cells be less than 5% of the ^3^H-thymidine incorporation of the non-irradiated cells. This criterion likely errs on the side of being too stringent, as cells with damaged chromosomes can incorporate ^3^H-thymidine to a limited extent even though they are incapable of proliferating, and some tumor cells damaged by radiation can continue to proliferate for a period of time before they die [38]. Thus, some vaccine preparations will not be used because of the inability to demonstrate the loss of proliferation in a short term assay. Alternatives to ^3^H-thymidine incorporation exist, but they are problematic as well. Trypan blue dye exclusion and counting is subject to a high rate of inter- and intra-operator error. Clonogenic assays are complicated by the fact that even in the absence of irradiation, only about 30–40% of all fresh melanoma samples yield a cell line (unpublished observations), thus lack of tumor outgrowth from a sample following irradiation doesn't necessarily reflect the results of irradiation. Likewise, xenografts into immunodeficient mice yield less than 100% outgrowth, and can require months to over a year for a tumor to become established [39].

When tumor cells from thirty-three melanoma patients were gamma-irradiated and subsequently tested for ^3^H-thymidine incorporation, eight of the preparations did not meet the release criteria (Figure 1). Five of these eight samples were among the nine samples with the lowest ^3^H-thymidine incorporation. This observation is consistent with that fact that cells do not always die immediately following radiation treatment but may first need to undergo several rounds of cell division as their genome becomes increasingly unstable [40]. Cells incorporating little ^3^H-thymidine are unlikely to be undergoing mitosis at a high rate and thus less likely to acquire the genomic instability necessary to lead to cell death. Although less often than cells with a low proliferative capacity, some samples with an initial high proliferative capacity clearly retained the ability to proliferate following gamma-irradiation. These results indicate that treatment with 200 Gy of gamma irradiation is not always sufficient to block the incorporation of ^3^H-thymidine into the DNA of melanoma samples, and that approximately 25% of the samples will fail a release criterion that requires the ^3^H-thymidine incorporation of the gamma-irradiated sample to be less than 5% of that obtained with the corresponding non-irradiated sample. Of the twenty-eight vaccine lots that were released for immunization, none resulted in outgrowth of the tumor at the site of immunization. Interestingly, while other studies have treated the autologous tumor to be used in vaccines with 25 to 200 Gy of irradiation [24-29], none of the studies apparently incorporated a release criterion demonstrating that the irradiation had substantially impacted on the ability of the cells to proliferate. Also, none of those studies reported outgrowth of the immunizing tumor at the injection site.

Although it is feasible to measure post-radiation ^3^H-thymidine incorporation capacity and to exclude patients from protocols whose tumors demonstrate incorporation after gamma-irradiation as was done for the Mel37 clinical trial, we wanted to develop a protocol that would more reliably block ^3^H-thymidine incorporation in the irradiated tumor cells. UV-irradiation, which is known to induce apoptosis [40-42], was therefore tested as an adjunct to gamma-irradiation.

Treatment of melanoma cell lines and patient samples with UV-irradiation (168 mJ/cm^2 ^UVA and 52 mJ/cm^2^) alone or in combination with gamma-irradiation (200 Gy) is more effective at reducing ^3^H-thymidine incorporation than is treatment with gamma-irradiation alone (Figure 3, 4, 5). This finding is consistent with the results of the cell viability assay which demonstrates that UV-irradiation either alone or in combination with gamma-irradiation induces a significant amount of death one day following treatment, while gamma-irradiation alone has no effect on viability at least through three days following treatment (Figure 7). Although in most cases UV-irradiation may be sufficiently effective on its own to reduce ^3^H-thymidine incorporation below that of 5% of the non-irradiated control, the combination of gamma-irradiation and UV-irradiation adds an extra layer of security in minimizing the possibility of tumor outgrowth following vaccination.

Gamma-irradiation can induce apoptosis in tumor cells [40,43,44], but 200 Gy of gamma-irradiation induced little to no apoptosis in the melanoma cell lines VMM39 and VMM86 when they were tested eight hours post-irradiation (Figure 6). This was true whether caspase was measured as an indicator of early apoptosis or DNA fragmentation was measured as an indicator of late apoptosis. Conversely, UV-irradiation, either alone or in combination with gamma-irradiation, induced apoptosis as measured by both the caspase and DNA fragmentation assays. Thus, the addition of UV-irradiation to the vaccine preparation protocol not only decreases the ^3^H-thymidine incorporation of the tumor cells such that it is more likely they will pass the release criteria, but it also enhances apoptosis which may increase the likelihood of uptake by dendritic cells with the subsequent presentation of tumor antigens [45].

A potential consequence of tumor cells beginning to undergo apoptosis and subsequent death is the degradation of cellular proteins by lysosomal proteases which could destroy antigens that would otherwise be acquired by dendritic cells. This in turn may prevent the subsequent cross-priming of T cells [46,47]. Therefore, we used immunohistochemistry to assess the expression of typical cellular proteins after gamma-irradiation and UV-irradiation (Table 1). Expression of gp100, MART-1/MelanA, and S100 was unaffected by gamma- or UV-irradiation. Conversely, tyrosinase expression was decreased by UV-irradiation alone or in combination with gamma-irradiation, but not by gamma-irradiation alone. T311, the antibody used to detect tyrosinase, binds to a linear epitope located within amino acids 233–247 of the protein [48]. Therefore, the most likely explanations for a loss of reactivity in immunohistochemistry is that the linear epitope has been cleaved and is no longer recognized by the antibody, or that the protein has been degraded to such an extent that proteolytic fragments containing the epitope no longer exist. To further investigate the possibility that the protein has been degraded we performed a Western blot analysis using the polyclonal antibody H-109 which was raised against amino acids 421–529 at the C-terminus of the tyrosinase protein (product insert). This analysis also showed that tyrosinase expression was greatly reduced in UV-irradiated cells. Taken together, these results suggest that the tyrosinase protein is not merely unfolded, but has undergone significant degradation. Should T cell stimulation occur as a result of recognition of processed peptide on the irradiated tumor cells rather than through cross priming on dendritic cells, it is possible that the loss of the epitope recognized by the antibody could be accompanied by an increase in peptide presentation by class I MHC molecules as the denatured/degraded protein may be processed more efficiently by proteasomes. These results indicate that additional studies are warranted to determine how irradiation, apoptosis, and necrosis affect antigen expression and the activation of antigen-specific T cells.

## Conclusion

When autologous melanoma cell vaccines are treated with 200 Gy of gamma-irradiation, 25% of them fail a lot release criterion requiring that ^3^H-thymidine incorporation of the irradiated samples be less than 5% of that seen in the non-irradiated controls. This release criterion could be met in all samples tested, however, when gamma-irradiation of the cells was supplemented with UV-irradiation. The results also indicate that UV-irradiation resulted in the degradation of at least one antigen in the treated cells, while preserving at least three other antigens. There are currently no standard lot release criteria for autologous melanoma vaccines, but we hope the current findings are informative toward future cell-based vaccine development.

## Competing interests

The authors declare that they have no competing interests.

## Authors' contributions

DHD, EMS, CED, ARC performed the experiments and reviewed/edited the manuscript. KTH analyzed and interpreted the data, and co-wrote the manuscript. KACB contributed to the conception of the experiments and reviewed/edited the manuscript. JWP and MWT performed the immunohistochemistry experiments and reviewed/edited the manuscript. CLS conceived the project and contributed to its design, analyzed and interpreted the data, and co-wrote the manuscript. All authors read and approved the final manuscript.

## Pre-publication history

The pre-publication history for this paper can be accessed here:


